# Probing the Dynamics of Doxorubicin-DNA Intercalation during the Initial Activation of Apoptosis by Fluorescence Lifetime Imaging Microscopy (FLIM)

**DOI:** 10.1371/journal.pone.0044947

**Published:** 2012-09-18

**Authors:** Nai-Tzu Chen, Chia-Yan Wu, Chao-Yu Chung, Yeukuang Hwu, Shih-Hsun Cheng, Chung-Yuan Mou, Leu-Wei Lo

**Affiliations:** 1 Division of Medical Engineering Research, National Health Research Institutes, Zhunan, Taiwan; 2 Department of Chemistry, National Taiwan University, Taipei, Taiwan; 3 National Synchrotron Radiation Research Center, Hsinchu, Taiwan; 4 Institute of Physics, Academia Sinica, Taipei, Taiwan; Biological Research Centre of the Hungarian Academy of Sciences, Hungary

## Abstract

Doxorubicin is a potent anthracycline antibiotic, commonly used to treat a wide range of cancers. Although postulated to intercalate between DNA bases, many of the details of doxorubicin’s mechanism of action remain unclear. In this work, we demonstrate the ability of fluorescence lifetime imaging microscopy (FLIM) to dynamically monitor doxorubicin-DNA intercalation during the earliest stages of apoptosis. The fluorescence lifetime of doxorubicin in nuclei is found to decrease rapidly during the first 2 hours following drug administration, suggesting significant changes in the doxorubicin-DNA binding site’s microenvironment upon apoptosis initiation. Decreases in doxorubicin fluorescence lifetimes were found to be concurrent with increases in phosphorylation of H2AX (an immediate signal of DNA double-strand breakage), but preceded activation of caspase-3 (a late signature of apoptosis) by more than 150 minutes. Time-dependent doxorubicin FLIM analyses of the effects of pretreating cells with either Cyclopentylidene-[4-(4-chlorophenyl)thiazol-2-yl)-hydrazine (a histone acetyltransferase inhibitor) or Trichostatin A (a histone deacetylase inhibitor) revealed significant correlation of fluorescence lifetime with the stage of chromatin decondensation. Taken together, our findings suggest that monitoring the dynamics of doxorubicin fluorescence lifetimes can provide valuable information during the earliest phases of doxorubicin-induced apoptosis; and implicate that FLIM can serve as a sensitive, high-resolution tool for the elucidation of intercellular mechanisms and kinetics of anti-cancer drugs that bear fluorescent moieties.

## Introduction

To be ability to monitor the intracellular dynamics of therapeutic agents is a challenging but pivotal of contemporary drug design. Doxorubicin, for example, is one of the most common and potent chemotherapeutic drugs used to treat cancer; demonstrating antitumor activity in a wide variety of malignancies that include lymphomas, leukemias, and solid tumors of the breast, pancreas, stomach, bladder, and ovaries.[1]–[3] While doxorubicin’s mechanism of action remains unclear, it is postulated to induce cellular apoptosis via drug-DNA intercalation, as evidenced in affected cells by double-strand breaks (DSBs) in DNA, [4] fragmented nuclei with condensed chromatin, and the formation of apoptotic bodies. While previous studies suggested that doxorubicin’s intercalation between DNA base pairs stabilizes the topoisomerase II cleavage complex, reports that other mechanisms – such as doxorubicin inducing early lipid peroxidation or DNA damage via activating ataxia-telangiectasia mutated (ATM)-dependnent phosphorylation through generation of reactive oxygen species – also contribute to doxorubicin-induced apoptosis.[5]–[8] A variety of approaches have been employed to elucidate the mechanism by which doxorubicin induces cell death. Using cell extracts, Tewey et al. found that administration of doxorubicin led to topoisomerase-II-mediated cleavage to DNA [8] while Frederick et al. mapped the three-dimensional structure of doxorubicin bound to DNA sequence d(CGATCG) using X-ray crystallography. [9] From these studies it was shown that doxorubicin inserted its planar chromophores between DNA bases, stabilized by hydrogen bonds, such that one of the rings acted as an anchor in the DNA’s minor groove. Such static, cell-free studies are, however, of limited value in determining the dynamics of doxorubicin-DNA interaction as they are unable to causally delineate the sequence of events in this process nor do they reflect the effects of the local environment.

High resolution microscopy of living cells suffers no such constraints. Fluorescence lifetime imaging microscopy (FLIM), in particular, can spatiotemporally monitor events of both cells and their microenvironment under physiological conditions with high precision. FLIM has been used to follow changes in the microenvironment of cells that endogenously express green fluorescence proteins (GFP) during the apoptosis, [10] as well to detect the temporal and spatial distributions of pH in cytoplasm and vesicular compartments of lysosomes via exogenous fluorescent probes. [11] Schneckenburger et al. developed 3D microscopic methods based on spectral imaging and FLIM to probe the intracellular interaction of intrinsic fluorescence molecules with their microenvironments [12] while others have combined FLIM with Förster resonance energy transfer (FRET) to monitor protein-protein interactions, molecule-DNA interactions, and molecule release dynamics from micro-particles.[13]–[16] In this study, we demonstrate the ability of FLIM to dynamically monitor doxorubicin-DNA intercalation during the earliest stages of apoptosis. Our studies demonstrate that FLIM can serve as a sensitive, high-resolution tool for the elucidation of intercellular mechanisms and kinetics of anti-cancer drugs that bear fluorescent moieties.

## Results and Discussion

**Figure 1:**
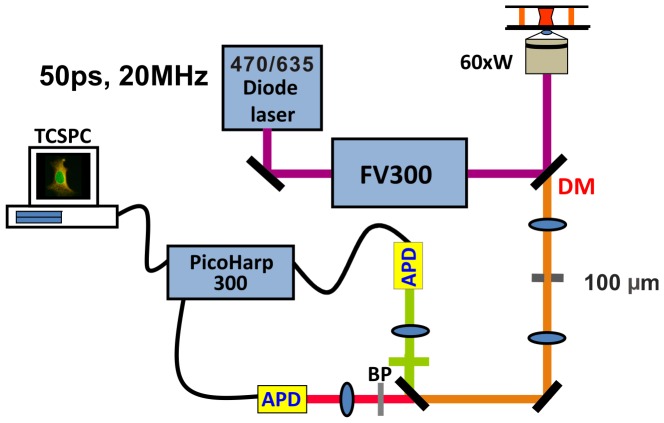
Diagram illustrating the principal components of the fluorescence lifetime imaging microscope. (Abbreviattion: DM, dichoroic mirrow; BP, bandpass filter; LP, longpass filter. Please refer the details in the FLIM section of Materials and Methods.

Doxorubicin diffusion and DNA targeting are difficult assess quantitatively using only fluorescence intensity measurements, as the complex scattering and absorption of light by various cellular structures tend to obscure the measured signal’s spatiotemporal content. By contrast, fluorescence lifetime measurements are relatively unaffected by photon scattering and absorption – providing, in addition to spatiotemporal data, information regarding the local milieu’s pH, temperature, and oxygenation. To best characterize the intracellular behavior of doxorubicin, we elected to record both fluorescence intensity and lifetime, using the apparatus shown in Figure 1.

**Figure 2:**
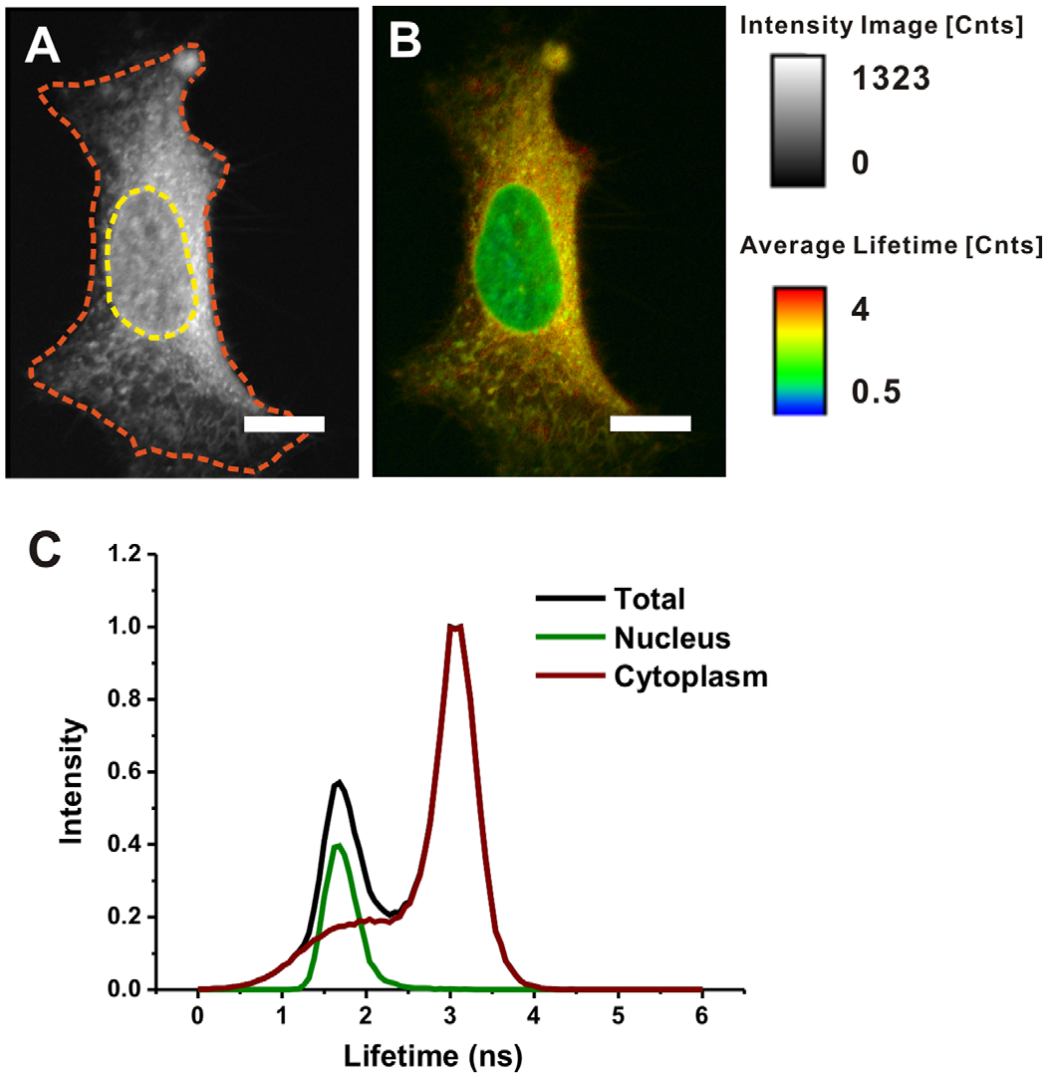
Fluorescence intensity, lifetime mapping, and corresponding lifetime histogram of doxorubicin in HeLa cells. Cells were treated 5 µg/ml doxorubicin for 24 hrs. (A) Fluorescence intensity image; the contours of nuclear envelop and cell membrane are delineated with yellow and red dotted lines respectively, based on the phase contrast image. (B) Fluorescence lifetime image. Scale bar, 10 µm. (C) Corresponding lifetime histogram of nucleus and cytoplasm.

Approximately 24 hrs after the addition of doxorubicin to plated HeLa cells, we optically excited samples at 488 nm and measured both their fluorescence intensities and lifetimes at wavelengths spanning 520–570 nm. As shown in the representative image Figure 2A, the spatial distribution of fluorescence intensities are typically well dispersed within the cytoplasm and nucleus, while the spatial distribution of fluorescence lifetimes (Figure 2B) appear much less homogeneous, suggesting the existence of several unique, doxorubicin-induced microenvironments. We then superimposed phase contrast images onto intensity/lifetime images, to delineate the contours of cell nuclei and cytoplasm via their corresponding nuclear envelopes and cell membranes. From such studies we generated, on a pixel-by-pixel basis, a fluorescence lifetime histogram whose maxima represent the average intracellular fluorescence lifetimes (τ_av_) of doxorubicin in HeLa cells: 1.68 ns within nuclei and 3.12 ns within cytoplasm (Figure 2C), as compared to 1.02 ns for an aqueous solution of doxorubicin.

**Figure 3:**
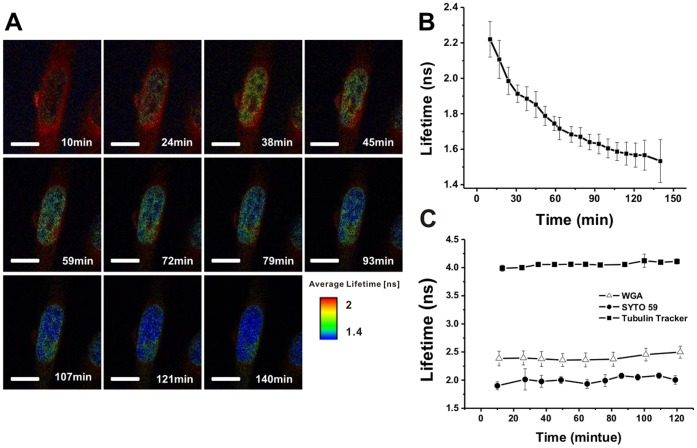
Dynamic monitoring of fluorescence lifetimes within nuclei. (A) Dynamic images of doxorubicin fluorescence lifetime in HeLa cells. (B) Changes in average fluorescence lifetime of doxorubicin in nuclei as a function of time. (C) Changes in average fluorescence lifetime of (i) (solid circle) an nucleus targeting dye, SYTO 59 in nucleus; (ii) (open triangle) an plasma membrance dye, WGA; (iii) (solid square) an tubulin labeling dye, Tubulintracker.

To examine drug dynamics within cells, we performed similar studies but via sequential imaging at 10–15 minute intervals, commencing 10–140 min after the addition of 5 µg/ml doxorubicin. Figure 3A shows a representative sequence of such images acquired for a single HeLa cell. Average fluorescence lifetimes of doxorubicin within nuclei were quantified as described above and plotted as a function of time. As shown in Figure 3B, doxorubicin’s average fluorescence lifetime decreased rapidly during the initial 2 hrs of treatment, suggesting a remodeling of the Doxorubicin-DNA intercalation site upon apoptotic initiation. To further substantiate our inferred correlation of doxorubicin-DNA intercalation with doxorubicin fluorescence lifetime, we employed an inert cell-permeant nucleic acid stain, SYTO 59, commonly used for nucleus staining of live cells. HeLa cells were seeded 24 hrs prior to the nucleus staining with 2 nM of SYTO 59 dye (excitation/emission: 622/645 nm). As illustrated in Figure 3C (solid circle), the fluorescence lifetime of SYTO 59 within nuclei was considerably more stable than that of doxorubicin; with the latter demonstrating a rapid and significant decrease during the corresponding time course (Figures 3A–B). For comparison, we also measured the fluorescence lifetimes of 2 cytoplasmic structure-specific dyes: Wheat Germ Agglutinin Fluor 488 Conjugates (WGA) (excitation/emission: 495/519 nm) and TubulinTracker (excitation/emission: 494/522 nm), for plasma membrane and tubulin labeling in live cells, respectively. Fluorescence lifetimes were monitored at 10–15 minute intervals following the addition of either 5 µg/ml WGA or 1 µM TubulinTracker to cells. As shown in Figure 3C (open triangle) and 3C (soild square), the fluorescence lifetimes of both dyes were stable for more than 120 mins following their administration, further supporting the notion that the observed variations in doxorubicin fluorescence lifetime were the result of DNA intercalation.

**Figure 4:**
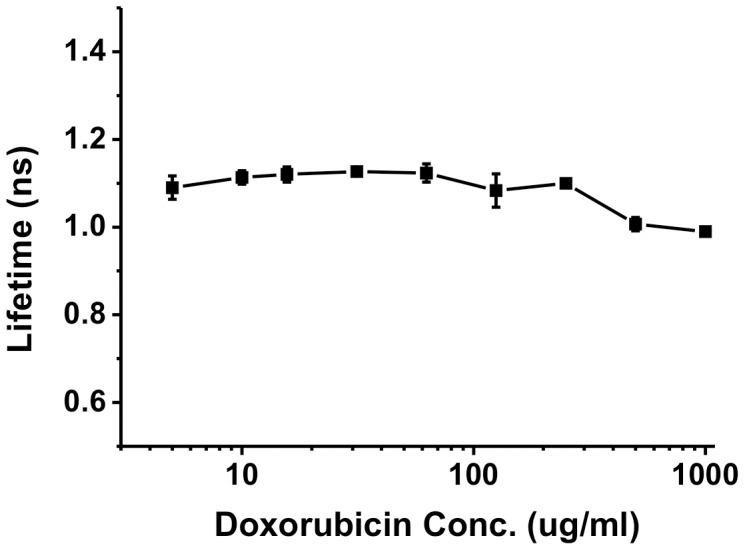
Fluorescence lifetime measuments at various concentrations of doxorubicin. Fluorescence lifetimes of doxorubicin were measured at various drug concentrations. Doxorubicin samples were prepared with MEM medium for 5 µg/ml to 1 mg/ml, while fluorescence lifetimes were measured by FLIM system.

To differentiate/eliminate fluorescence quenching effects from observed fluorescence lifetime measurements at high doxorubicin concentrations, we recorded the fluorescence lifetime decays of various drug concentrations in cell culture media. The fluorescence flux (photon count) from nuclei at 150 mins was ∼20 times greater than that at 10 mins (data not shown), suggesting that doxorubicin had reached at least 20 times higher concentration in nuclei during the same period (without regard to any potentially existent, fluorescence self-quenching). Accordingly, we measured the fluorescence lifetime of doxorubicin from 5 µg/ml to 1 mg/ml in culture media, the result of which is summarized in Figure 4. The fluorescence lifetime of doxorubicin remained stable at 1.1 ns from 5 µg/ml to 250 µg/ml (50 times increase of concentration) and slightly decreased to 1.01 ns at 500 µg/ml (100 times increase of concentration). These studies indicate that a 50x increase in Doxorubicin concentration does not alter the fluorescence lifetime of doxorubicin, while 100x increase in doxorubicin concentration merely garnered a 0.09 ns decrease in lifetime. Taken together, these data demonstrate that the observed decrease of doxorubicin fluorescence lifetime in cell nuclei arose neither from static self-quenching or Förster resonance energy transfer (FRET)-related phenomena.

**Figure 5:**
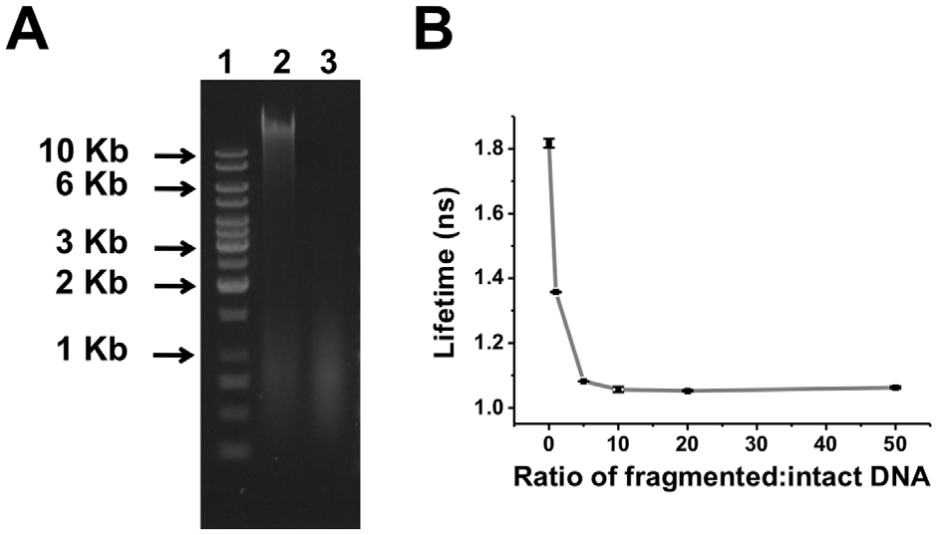
Fluorescence lifetime measurements of doxorubicin for various ratios of fragmented/intact DNA. (A) Gel electrophoresis analysis of intact and fragmented DNA; Line1: 1 Kb DNA ladder, Line2: Genomic DNA extracted from HeLa cells, Line3: Genomic DNA digested by DNase I (1 unit/µl for 1 hr). (B) Fluorescence lifetime of doxorubicin at increasing ratios of fragmented/intact DNA at a fixed ratio of DNA/doxorubicin by weight.

To determine if apoptosis-induced remodeling of chromatin structures altered the fluorescence lifetime of doxorubicin, genomic DNA from HeLa cell was extracted and digested with DNase I, to mimic the microenvironment of DNA laddering – a phenomenon specific to apoptosis. The size of intact and fragmented DNA samples were analyzed using conventional agarose gel electrophoresis (Figure 5A). Alongside the 1 Kb DNA ladder (lane 1), genomic DNA extracted from HeLa cells (lane 2) revealed its primary component to be larger than 10 Kb – with a small amount of DNA distributed around 1 Kb reflecting extraction-related fragmentation. By contrast, genomic DNA after DNase I digestion (lane 3) exhibited a continuous distribution around 1 Kb, reflecting its complete fragmentation. Fluorescence lifetimes were then measured in solutions containing 5 µg/ml doxorubicin and various ratios of fragmented:intact DNA (ratios ranged from pure/intact DNA (0∶1) to 50∶1). For solutions with a fixed 50∶1 ratio of DNA:doxorubicin (by weight), we measured the fluorescence lifetimes of doxorubicin with increasing ratios of fragmented:intact DNA. The total concentration of DNA (fragmented + intact) was held constant, to eliminate variations in the fluorescence lifetime distribution of free/bound DNA. As shown in Figure 5B, the fluorescence lifetime of doxorubicin decreased rapidly from 1.81 ns to 1.05 ns in going from pure DNA to the ratio of 10∶1 fragmented:intact DNA, and became invariant as the fragmented:intact DNA ratio exceeded 10∶1. These findings suggest that variations in DNA microenvironment can significantly modulate the fluorescence lifetime of doxorubicin.

**Figure 6:**
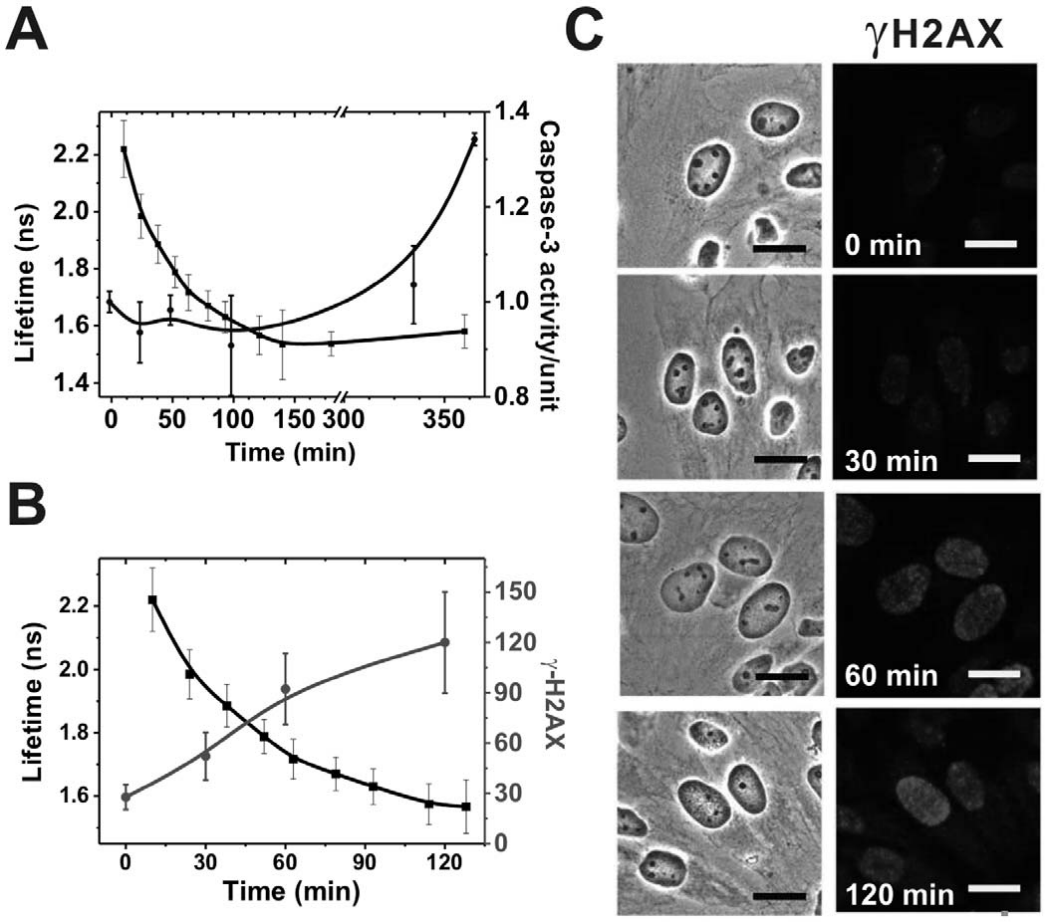
Doxorubicin induces caspase-3 activation and H2AX phosphorylation. HeLa cells were exposed to 5 µg/ml doxorubicin for different periods, after which cell lysates of each time point were extracted for either measurement of caspase-3 activity or fixed for the γH2AX staining. Doxorubicin fluorescence lifetimes following treatment (black) vs. (A) caspase-3 activity (blue); and (B) γH2AX activation (red). γH2AX activation was quantified from confocal microscopic images, as shown in (C), using MetaMorph imaging processing software. As illustrated, caspase-3 activation is preceded by the H2AX phosphorylation. (Scale bars in C: 20 µm).

To correlate the change of doxorubicin fluorescence lifetime with the apoptotic cascade in live cells, activity assays of the biological effectors caspase-3 and γH2AX were concurrently employed. Among apoptosis effectors, caspase-3 has been identified as being a key mediator of the execution phase of apoptosis in mammalian cells. [17] HeLa cells were incubated with 5 µg/ml of doxorubicin in medium for 0, 30, 60, 120, 300, 360 mins and extracted for caspase-3 protein activity analysis. Figure 6A shows the time-dependence, post drug administration, of both the relationship between temporal profile of caspase-3 activation (blue) and as compare with the fluorescence lifetime of doxorubicin (black) for an incubation period of 120 min. As illustrated, caspase-3 became activated approximately 120 mins after the addition of doxorubicin to cells, whereas doxorubicin’s fluorescence lifetime began to decrease almost immediately following drug addition. Thus measurements of fluorescence lifetimes provided a much earlier indicator of doxorubicin-induced apoptosis than did measurements of caspase-3 activation.

H2AX, a variant of the core histone protein H2A, accounts for 2–25% of total H2A cellular content. As H2AX phosphorylation (γH2AX), in response to DNA double-strand breaks (DSBs), is known to be a sensitive indicator of early-stage apoptosis. We incubated HeLa cells with 5 µg/ml of doxorubicin for periods of 30–120 mins, then immuno-stained cells with γH2AX antibodies and conducted confocal microscopy (Figure 6C). [4], [18], [19] Average fluorescence intensities of activated γH2AX were then determined and plotted as a function of doxorubicin fluorescence lifetimes during the first 120 mins following doxorubicin administration. As Figure 6B illustrates, doxorubicin-induced γH2AX activation is concurrent with decreases in doxorubicin’s fluorescence lifetime during the initial phase of apoptosis – quite unlike the 120 mins delay observed with caspase-3 activation (Figure 6A).

**Figure 7:**
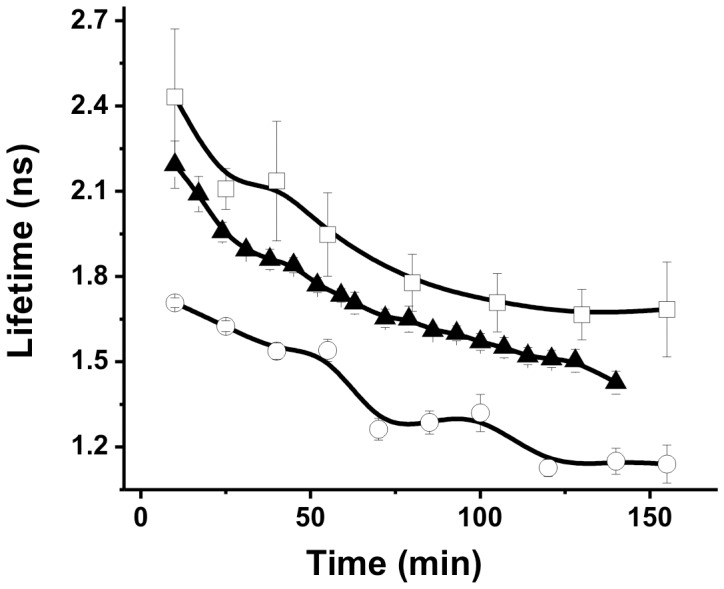
Temporal profiles of doxorubicin fluorescence lifetime in nuclei after CPTH_2_ and TSA treatments. CPTH_2_ (open square) and TSA (open circle) were added to culture medium 24 hrs before cells were exposed to 5 µg/ml doxorubicin. 5 µg/ml doxorubicin (solid triangle) served as the control.

Equilibrium dialysis, hydrodynamic analysis, and electric dichroism have shown that anthracycline antibiotics, including dorubicin, typically bind the linker region of chromatin and alter the linker twist. These bindings not only change the orientation of adjacent nucleosomes but also lead to chromatin unfolding and aggregation. [20]–[22] Such drug-induced structural changes may also ultimately contribute to apoptosis. Chromatin structures are majorly altered by acetylation and deacetylation on amino termini of core histone proteins which are regulated by two enzymes: histone acetyltransferase (HAT) and histone deacetylase (HDAC). To further characterize Doxorubicin’s role in potentially altering chromatin structure/function, several molecular inhibitors of chromatin remodeling-related proteins were employed: Trichostatin A (TSA: an inducer of chromatin decondensation and inhibitor of HDAC) [23] and Cyclopentylidene-[4-(4-chlorophenyl)thiazol-2-yl)-hydrazine (CPTH_2_: an inducer of chromatin condensation and inhibitor of HAT). [24] 400 nM TSA or 100 µg/ml CPTH_2_ was added to HeLa cell cultures 24 hrs prior to their 5 µg/ml doxorubicin administration. Following doxorubicin addition, fluorescence lifetime measurements were made to quantify the effects of TSA and CPTH_2_ on HDAC and HAT, respectively. Earlier studies have shown that TSA-induces a global increase in histone acetylation and leads to chromatin decondensation 24 hrs post administration, [25]–[26] whereas CPTH_2_ specifically inhibits GCN5-dependent acetylation on histone H3 K14, resulting in chromatin condensation and consequently the slow growth rate of yeast. [24] [27] Therefore, it is concluded that the chromatin structure is decondensed following TSA treatment whereas is condensed after CPTH_2_ treatment. Figure 7 shows the time-dependent fluorescence lifetimes of doxorubicin-untreated cells relative to time-dependent fluorescence lifetimes of cells previously treated with one of these two chromatin remodeling inhibitors. In the first 150 mins following their respective additions of doxorubicin, fluorescence lifetimes of TSA-treated cells decreased from 1.70 ns to 1.14 ns while lifetimes of CPTH_2_-treated cells decreased from 2.43 ns to 1.68 ns – as compared to 2.23 ns to 1.48 ns lifetime decrease observed in cells not treated with chromatin remodeling inhibitors. Despite the initial offset in fluorescence lifetimes resulting from pretreatments with either TSA or CPTH_2_, the similarity in shape of one curve to another in Figure 7 reflects that the initial condensation/decondensation staus of chromatin affects the doxorubicin’s fluorescence lifetime but imposes no acceleration/deceleration of the following action of doxorubicin on chromatin. When viewed in the context of the electrophoresis studies described earlier, these findings suggest that doxorubicin fluorescence lifetime measurements can be used to sense very subtle changes in the remodeling of chromatin structure prior to the inauguration DNA fragmentation.

### Conclusion

In this work we demonstrate for the first time the applicability of fluorescence lifetime imaging microscopy (FLIM) to monitor the dynamics of doxorubicin-DNA intercalation during the earliest stages of doxorubicin-induced apoptosis. By monitoring the rate of change of doxorubicin fluorescence lifetimes in nuclei, one can detect very subtle changes in chromatin structure well in advance of the commencement of DNA fragmentation. As such, FLIM can serve as a sensitive, high-resolution tool for the elucidation of intercellular mechanisms and kinetics of anti-cancer drugs that bear fluorescent moieties.

## Materials and Methods

### Fluorescence Lifetime Imaging Microscopy (FLIM)

Figure 1 shows the experimental apparatus used in our studies. Single-photon, time-domain FLIM experiments were performed using an inverted microscope (Olympus IX71, Tokyo, Japan) illuminated by a 470/635 nm diode laser (Picoquant GmbH, Berlin, Germany) generating 50 picosecond pulses at a repetition rate of 20 MHz. Coherent light from the laser diode was first passed through a polarization-maintaining, single-mode optical fiber, to provide a Gaussian beam profile. The shaped beam was then reflected by a dichroic mirror (F53-470, AHF Analysentechnik AG) and scanning galvanometer (Olympus FV300, Tokyo, Japan) prior to its introduction to the inverted microscope. A 60x water immersion objective of 1.2 numerical aperture (NA) (Olympus, Tokyo, Japan) was used both to deliver the excitation beam and to collect fluorescence emission, while a 100 µm diameter confocal pinhole was used to reject out of focus fluorescence. A longpass filter (HQ500LP, Chroma Technology) and bandpass filter (FF01-550/88, Semrock) were used to exclude excitation light from admission to the detector: a high-speed avalanche photodiode (APD) (Micro Photon Devices, Bolzano, Italy). The APD’s TTL output and laser diode trigger were passed through a time-correlated, single photon counting (TCSPC) module (PicoHarp300, Picoquant GmbH, Berlin, Germany). Subsequent data analysis and image processing were performed with Symphotime software 5.0 (Picoquant GmbH, Berlin, Germany).

### Cell Culture and Cell Uptake

HeLa cells were purchased from American Tissue Culture Collection (ATCC). The cells were cultured in a humidified, 37°C atmosphere comprised of 5% CO_2_ and 95% room air. Cell culture medium, Minimum Essential Medium (MEM; Gibco), was supplemented with 10% fetal bovine serum (FBS; Hyclone). For fluorescence lifetime measurements, cells were plated 24 hrs before each experiment and then given 5 µg/ml Doxorubicin immediately prior to imaging.

### Analysis of Caspase-3 Activity

Caspase-3 activity was detected using a Caspase-3 Colorimetric Activity Assay Kit (Chemicon). Briefly, HeLa cells were seeded into 35 mm plates at 1×10^5^ cells/well. After being exposed to doxorubicin for indicated number of minutes, cells were washed with PBS and retrieved by harvest solution (40 mM Tris-HCl pH 7.4, 1 mM EDTA, 150 mM NaCl). Cell lysates were then extracted using a conventional lysis buffer (PBS+0.1% NP-40) and the protein content determined using a BIO-RAD Bradford protein assay. Each 96-well microplate well received 70 µl cell lysate, 20 µL assay buffer, and 10 µL caspase-3 substrate (Ac-DEVD-pNA). Reactions were incubated at 37°C for 2 hrs and detected with a microtiter plate reader operating at 405 nm.

### Detection of γH2AX

To evaluate H2AX phosphorylation, HeLa cells were first seeded into an eight-well chamberslide (BD Falccon) at 1×10^5^ cells/well 24 hrs before drug treatment. 5 µg/ml doxorubicin were added to each well for 30, 60 and 90 mins incubations. Cells were then washed with PBS and fixed in 4% (w/v) paraformaldehyde for 15 mins at room temperature, followed by permeabilization in 0.25% (v/v) Triton X-100 for 30 mins. After permeabilization, slides were blocked with 5% BSA (bovine serum albumin) for 60 mins prior to their incubation with mouse monoclonal anti-γH2AX antibodies (1∶500). After washing with PBS, slides were incubated with Dylight (ex 654 nm, em 673 nm)-conjugated goat-anti-mouse secondary antibodies (1∶5000) for 60 mins, followed by additional PBS washings. Cell nuclei were counterstained with DAPI (1 µg/ml in PBS) for 10 mins. After an additional wash, slides were mounted with coverslips and viewed using an Olympus FV10i confocal microscope. Quantitative image analyses were conducted using MetaMorph software (Manufacturer, City/Country).

### *In vitro* Lifetime Detection

*In vitro* fluorescence lifetimes of various concentrations of doxorubicin were measured in solution under 470 nm diode laser excitation. Doxorubicin was prepared in concentrations of 5 µg/ml to 1 mg/ml in MEM medium. For fluorescence lifetime measurements of doxorubicin-DNA intercalation, genomic DNA was first extracted from 5×10^6^ HeLa cell samples using a Blood and Tissue Genomic DNA Extraction Kit (Viogene). The fragmented genomic DNA was the reacted (100 µl total volume) with 1 unit/µl DNase I in 1x reaction buffer (10 mM Tris-HCl (pH 7.5), 2.5 mM MgCl_2_, 100 µM CaCl_2_) at 37°C for 1 hr, after which the reaction was stopped by heat inactivation at 75°C for 10 mins. Verification and quantification of intact DNA and fragmented DNA were made via 1.5% agarose gel electrophoresis. Doxorubicin fluorescence lifetimes were measured in solutions containing 5 µg/ml doxorubicin and varying amounts of fragmented/intact DNA (with ratios ranged from pure/intact DNA to 50∶1). For solutions with a fixed 50∶1 ratio of DNA:doxorubicin (by weight), we measured the fluorescence lifetime of doxorubicin for increasing ratios of fragmented:intact DNA.

### *In vitro* Cell Lifetime Detection

For *in vitro* fluorescence lifetime measurements, cells were plated 24 hrs before each experiment and then given 5 µg/ml doxorubicin immediately prior to imaging. Various fluorescence dyes for labeling different cell organelles were employed for landmarking. SYTO 59 (excitation/emission: 622/645 nm, Molecular Probes, Inc.), Wheat Germ Agglutinin Fluor 488 Conjugates (excitation/emission: 495/519 nm, Invitrogen) and TubulinTracker™ (excitation/emission: 494/522 nm, Molecular Probes, Inc.) were employed to delineate nucleus, plasma membrane and tubulin, respectively. Fluorescence lifetime images were acquired as a function of time using the FLIM system described above with 470 nm or 635 nm laser excitation. For each experiment, Regions of Interest (ROIs) within more than 10 cells were selected and quantitated for each labeling dye. The nuclear regions of doxorubicin treated cells were delineated via phase contrast image superposition. HeLa cells whose chromatin structure were intentionally altered, were pretreated with either 400 nM TSA or 100 µg/ml CPTH_2_) 24 hrs prior to their use, and their medium replaced with complete medium containing 5 µg/ml doxorubicin.
