# Correction: Probing the Dynamics of Doxorubicin-DNA Intercalation during the Initial Activation of Apoptosis by Fluorescence Lifetime Imaging Microscopy (FLIM)

**DOI:** 10.1371/annotation/4c43c8c8-0a4e-425b-a72f-74e84f6f3c28

**Published:** 2012-11-05

**Authors:** Nai-Tzu Chen, Chia-Yan Wu, Chao-Yu Chung, Yeukuang Hwu, Shih-Hsun Cheng, Chung-Yuan Mou, Leu-Wei Lo

There are formatting errors in Figure 6. A correct image of Figure 6 can be seen here: 

**Figure:**
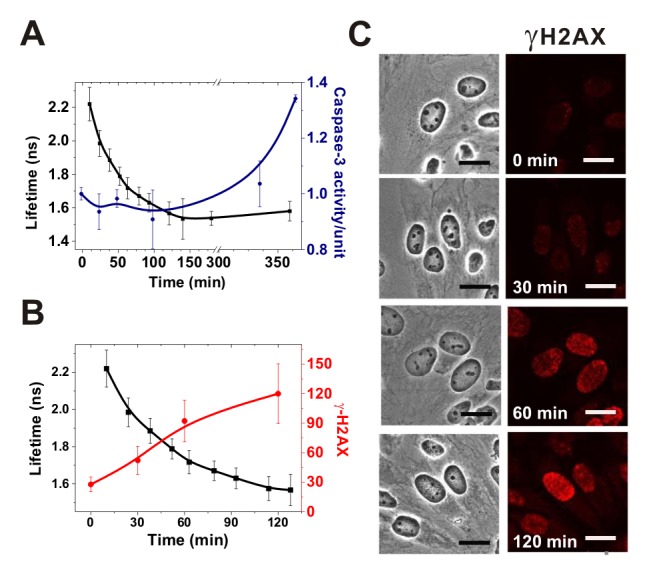


[^] 

